# Effects of an Adipose Mesenchymal Stem Cell-Derived Conditioned medium and TGF-β1 on Human Keratinocytes In Vitro

**DOI:** 10.3390/ijms241914726

**Published:** 2023-09-29

**Authors:** Hyrije Ademi, Katarzyna Michalak-Micka, Ueli Moehrlen, Thomas Biedermann, Agnes S. Klar

**Affiliations:** 1 Tissue Biology Research Unit, Department of Surgery, University Children’s Hospital Zurich, 8952 Schlieren, Switzerland; 2 Children’s Research Center, University Children’s Hospital Zurich, 8032 Zurich, Switzerland; 3 Faculty of Medicine, University of Zurich, 8032 Zurich, Switzerland; 4 Department of Surgery, University Children’s Hospital Zurich, 8032 Zurich, Switzerland

**Keywords:** keratinocyte, adipose-derived stem cells (ASCs), TGF-β1, wound healing, ASC secretome, Notch signaling, basal/suprabasal keratinocyte markers, epithelial differentiation markers

## Abstract

Human keratinocytes play a crucial role during skin wound healing and in skin replacement therapies. The secretome of adipose-derived stem cells (ASCs) has been shown to secrete pro-healing factors, among which include TGF-β1, which is essential for keratinocyte migration and the re-epithelialization of cutaneous wounds during skin wound healing. The benefits of an ASC conditioned medium (ASC-CM) are primarily orchestrated by trophic factors that mediate autocrine and paracrine effects in keratinocytes. Here, we evaluated the composition and the innate characteristics of the ASC secretome and its biological effects on keratinocyte maturation and wound healing in vitro. In particular, we detected high levels of different growth factors, such as HGF, FGFb, and VEGF, and other factors, such as TIMP1 and 4, IL8, PAI-1, uPA, and IGFBP-3, in the ASC-CM. Further, we investigated, using immunofluorescence and flow cytometry, the distinct effects of a human ASC-CM and/or synthetic TGF-β1 on human keratinocyte proliferation, migration, and cell apoptosis suppression. We demonstrated that the ASC-CM increased keratinocyte proliferation as compared to TGF-β1 treatment. Further, we found that the ASC-CM exerted cell cycle progression in keratinocytes via regulating the phases G1, S, and G2/M. In particular, cells subjected to the ASC-CM demonstrated increased DNA synthesis (S phase) compared to the TGF-β1-treated KCs, which showed a pronounced G0/G1 phase. Furthermore, both the ASC-CM and TGF-β1 conditions resulted in a decreased expression of the late differentiation marker CK10 in human keratinocytes in vitro, whereas both treatments enhanced transglutaminase 3 and loricrin expression. Interestingly, the ASC-CM promoted significantly increased numbers of keratinocytes expressing epidermal basal keratinocyte markers, such DLL1 and Jagged2 Notch ligands, whereas those ligands were significantly decreased in TGF-β1-treated keratinocytes. In conclusion, our findings suggest that the ASC-CM is a potent stimulator of human keratinocyte proliferation in vitro, particularly supporting basal keratinocytes, which are crucial for a successful skin coverage after transplantation. In contrast, TGF-β1 treatment decreased keratinocyte proliferation and specifically increased the expression of differentiation markers in vitro.

## 1. Introduction

The epidermis represents the outermost barrier of the human skin, protecting against external factors, and plays an important role in wound healing. Keratinocytes (KCs) are the main cell type present in the epidermis; therefore, their role is crucial for proper skin homeostasis. To renew the epidermis, KCs proliferate and differentiate from the basal layer upwards, until, in the uppermost viable cells, they dehydrate and flatten into corneocytes [1]. This uppermost epithelial layer of the skin is also referred to as the *stratum corneum* [2]. During wound healing, KCs re-epithelialize the injured area by migrating and proliferating at the wound site to restore the epidermis. Multiple factors, such as cytokines and growth factors, modulate this process [1].

Transforming growth factor-β (TGF-β) plays a crucial role in skin homeostasis and wound healing. In mammals, TGF-β consists of three different isoforms (TGF-β1, TGF-β2, and TGF-β3), which show a specific expression pattern throughout the epidermal layers [3]. TGF-β modulates epidermal homeostasis by regulating proliferation and differentiation in unwounded skin. However, upon injury, TGF-β factors induce KC migration [4]. Additionally, TGF-β is believed to regulate re-epithelialization, inflammation, angiogenesis, and granulation [4], therefore playing a crucial role in wound healing in general.

Recently, the TGF-β pathway, originally perceived as a linear, non-amplified pathway, has been shown to be more complex through its multi-step activation. When secreted, the TGF-β pro-peptide remains latent, allowing to build-up inactive TGF-β in the extracellular matrix (ECM), the so-called latency-associated peptide prodomain (LAP) [5].

Specifically, the TGF-β1 isoform, which is localized in the upper differentiated epidermal layers [6], acts as a growth inhibitory cytokine by arresting the cell cycle in the G1 phase [4]. However, during skin wound healing, TGF-β1 was reported to promote KC migration to the wound site by stimulating matrix metalloproteinases (MMPs), which work together with other components of the ECM to promote KC proliferation to close the wound [1]. Therefore, TGF-β1 is a crucial regulator of KC proliferation and myofibroblast differentiation during wound healing.

Further, Blokzijl *et al.* reported a cross-talk between the TGF-β pathways and Notch based on protein–protein interactions with the intracellular domain of Notch1 (NICD) and the Smad3 protein in vitro [7]. Notch is a family of cell surface receptors that regulate skin morphogenesis and skin homeostasis, as well as wound healing. It is a known tumor suppressor factor inhibiting the skin inflammatory response [8]. The Notch pathway activation regulates skin homeostasis by balancing the growth arrest and differentiation processes of KCs [9]. Specific Notch ligands found in the epidermis are Jagged1, Jagged2, and DLL1 [10], where Jagged1 expression is specifically observed in the suprabasal layers [11], while Jagged2 and DLL1 are restricted to the basal layer [12]. Through cell–cell interactions, Notch communicates with neighboring KCs in the human epidermis by promoting KC differentiation [8]. Later, Xu *et al*. described that Notch1 and TGF-β are both required to increase the concentrations of receptor protein tyrosine phosphatase-κ (PTPRK), which dephosphorylates epidermal growth factor receptor (EGFR), therefore down-regulating the cell proliferation of human KCs [13].

Interestingly, we previously reported an increased secretion of TGF-β1 in mesenchymal stem cells in adipose tissue, known as the so-called adipose tissue-derived stem cells (ASCs), as compared to dermal fibroblasts [14].

In fact, increased concentrations of TGF-β1 in the ASC secretome contribute significantly to the immunomodulatory properties of those cells [15]. Interestingly, in this study, we show that the secretome of ASCs contains several pro-healing factors, including keratinocyte growth factor (KGF), hepatocyte growth factor (HGF), epidermal growth factor (EGF), members of the vascular endothelial growth factor (VEGF) family, basic fibroblast growth factor (FGFb), platelet-derived growth factor-BB (PDGF-BB), insulin-like growth factor-1 (IGF-1), and key enzymes, such as matrix metalloproteinase-9 (MMP-9), among others [16,17]. The ASC-secreted TGF-β1 activates the KCs and fibroblasts of the skin and facilitates KC migration, proliferation, and differentiation [4].

The goal of this study was to assess the impact of the ASC secretome and/or TGF-β1 on primary human KCs in vitro. Using these two specific treatments, we observed significant differences in KC phenotype and in the expression of proliferation, differentiation, and stem cell markers involved in the Notch signaling pathway.

## 2. Results

### 2.1. Phenotypic Characterization of ASCs

**Figure 1:**
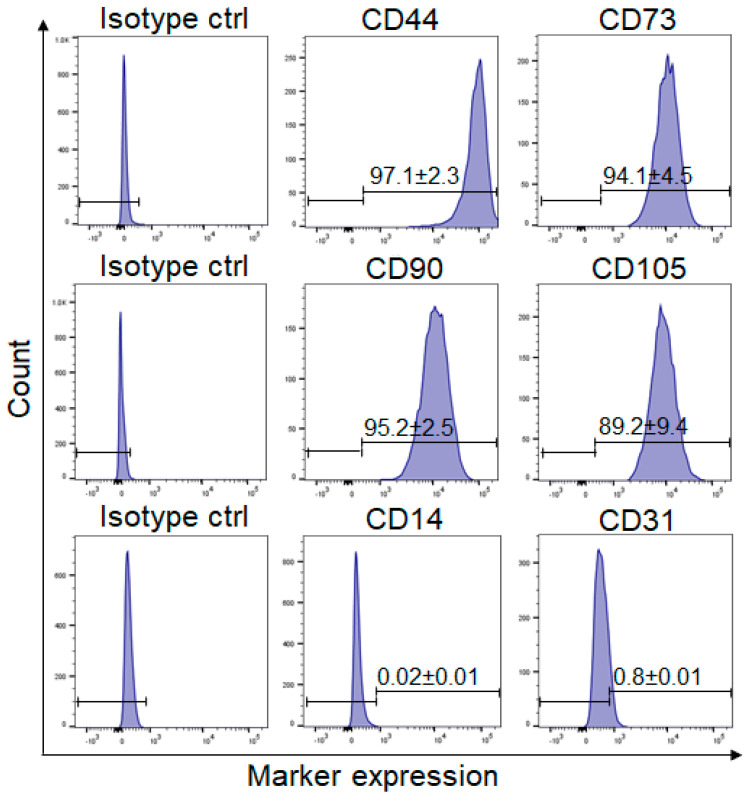
Flow cytometry analysis of stem cell markers expression on ASCs. ASCs express CD73, CD44, CD90, and CD105, while being negative for CD14 and CD31. Isotype-matched antibodies were used as a negative control. Representative histograms from 3 different donors are shown (*n* = 3).

In order to characterize and confirm the identity of the isolated adipose-derived stem cells (ASCs), different stem cell markers were analyzed through flow cytometry (Figure 1). In accordance with the minimal required expression criteria proposed by the International Society for Cellular Therapy (ISCT) [18], ASCs (*n* = 3) expressed CD44 (97.1 ± 2.3%), CD73 (94.1 ± 4.5%), CD90 (95.2 ± 2.5%), and CD105 (89.2 ± 9.4%), but were negative for CD14 (0.02 ± 0.01%) and CD31 (0.8 ± 0.01%) (Figure 1).

### 2.2. Trilineage Differentiation Potential of ASCs

**Figure 2:**
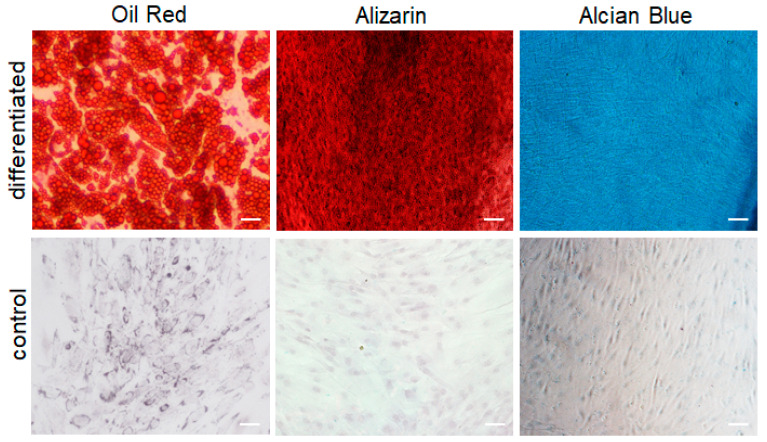
Representative pictures of trilineage differentiation staining. Upper row left: Oil Red O staining for adipogenic differentiation, upper middle: alizarin staining for osteogenic differentiation, and upper right: alcian blue staining for chondrogenic differentiation. Bottom row: corresponding control (control). Scale bar: 50 µm (*n* = 3 independent biological donors).

To assess the trilineage differentiation potential of ASCs (*n* = 3 independent donors), they were differentiated into adipocytes, osteocytes, and chondrocytes in vitro (Figure 2, upper row). Accordingly, Oil Red O staining confirmed adipogenic differentiation, alizarin staining osteogenic differentiation, and alcian blue staining chondrogenic differentiation of the ASCs. Cells maintained in a regular culture medium showed no differentiation (Figure 2, bottom row).

### 2.3. Viability of TGF-β1- and ASC-CM-Treated Human KCs In Vitro

**Figure 3:**
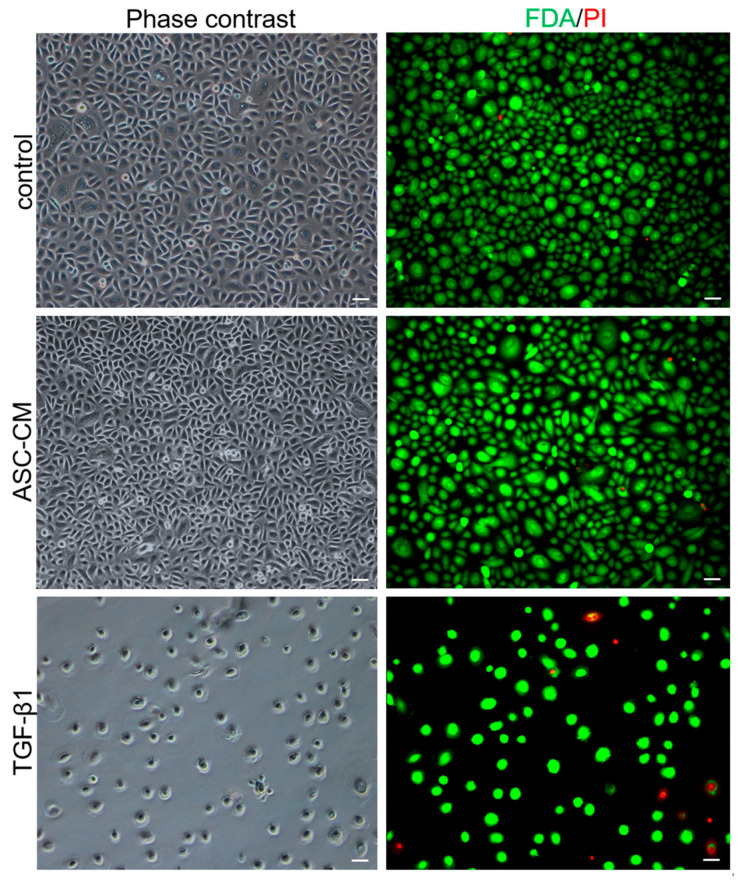
Phase contrast and live/dead stainings of human KCs. KCs were cultured in standard culture media (untreated: control), ASC-CM, or media with TGF-β1. Green FDA staining indicates live cells, whereas PI was used to detect dead cells (red). The KCs (*n* = 3) show no noticeable change over the course of 72 h in the ASC-CM (*n* = 3) compared to untreated KCs. In contrast, TGF-β1-treated KCs show less proliferation compared to untreated KCs. Images were taken after 72 h of in vitro culture. Shown are representative images of the three different treatments. Scale bars = 50 μm.

Phase contrast images and fluorescein diacetate (FdA)/propidium iodide (PI) staining of untreated and ASC-CM-treated KCs (*n* = 3 independent donors) demonstrated a similar morphology and cell numbers after 72 h of treatment (Figure 3). In contrast, stimulation with TGF-β1 led to impaired KC proliferation, as observed via reduced FdA and increased PI staining compared to the untreated (control) and ASC-CM-treated KCs (Figure 3).

### 2.4. Migration Potential of KCs Following ASC-CM and TGF-β1 Treatment

**Figure 4:**
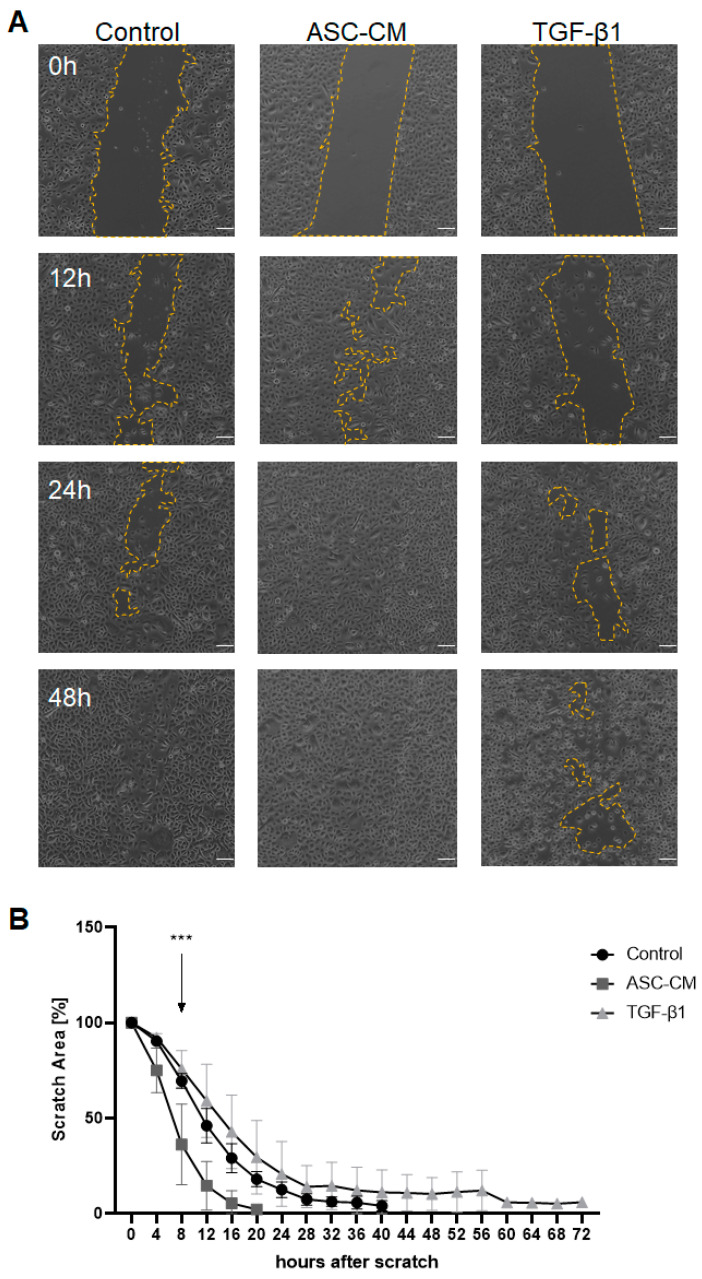
Migration potential of KCs treated with ASC-CM or TGF-β1. (**A**) Phase contrast images of human KCs cultured either in standard culture media (control), ASC-CM, or media with TGF-β1 after the scratch was performed. Migration was tracked for 72 h. Yellow lines: scratch area. Scale bars = 100 μm. (**B**) Quantification of scratch area every 4 h. After 8 h there is a significant decrease in the scratch area (*** *p* < 0.001) of the KCs treated with the ASC-CM compared to the control and TGF-β1-treated KCs. Full closure of the scratch area was detected at 20 h for ASC-CM-treated KCs, and at 40 h for control KCs. Regarding the TGF-β1-treated KCs, no full closure of the scratch area could be observed at the end of the experiment. However, a morphology change of the cells turning into roundish, detaching KCs was observed in this group. *n* = 3 independent donors. The graph represents the mean ± SD percentage of scratch area. Statistical analysis: two-way ANOVA (*** *p* < 0.001).

In addition to the viability assay, an in vitro migration assay showed that treatment with the ASC-CM increased the migration potential of KCs. We observed that after 20 h the scratch area was fully closed, whereas the control group required 40 h to close the wound. TGF-β1-treated KCs were not able to fully close the scratch area, even after 72 h, and they changed their morphology to roundish cells during this process (Figure 4).

### 2.5. Cell Cycle Analysis

**Figure 5:**
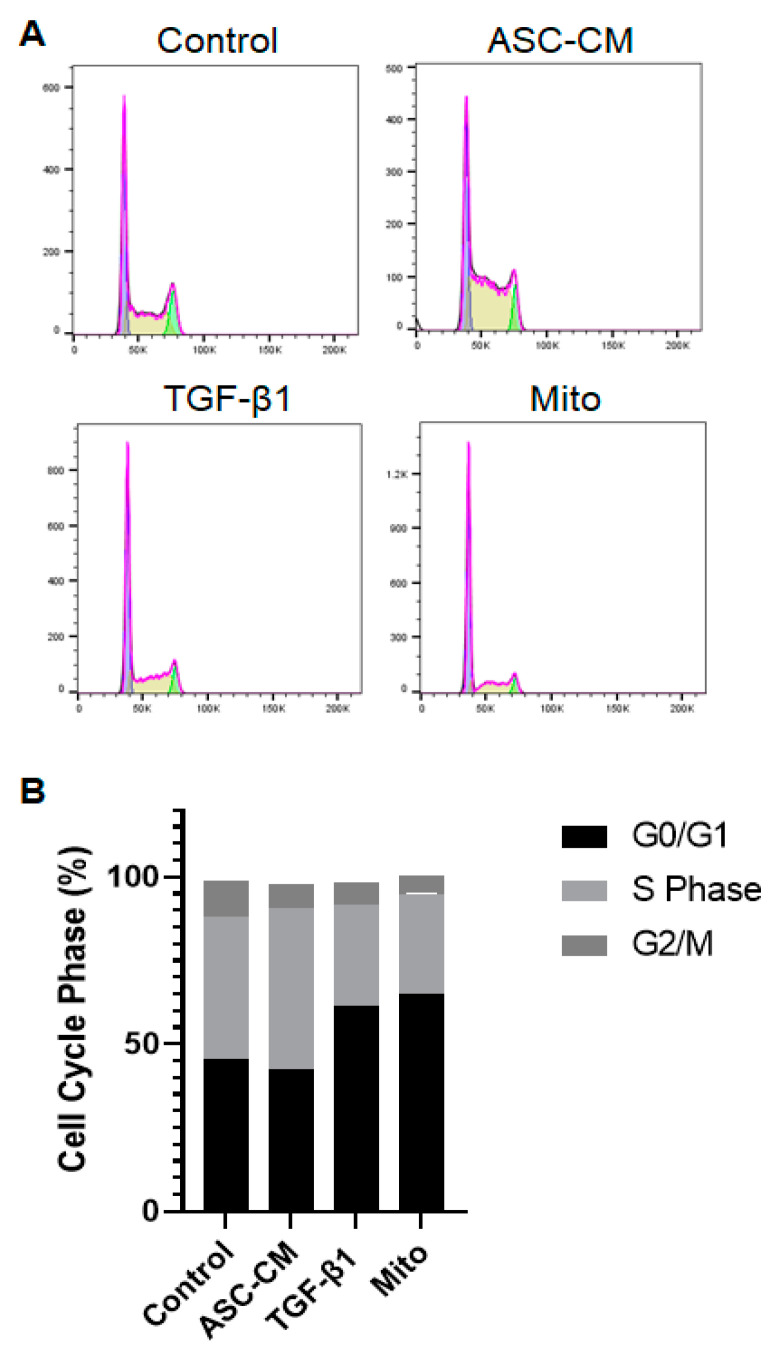
Cell cycle analysis of KCs. The cell cycle was detected through flow cytometry using PI staining. KCs were treated with ASC-CM, TGF-β1, or left untreated (control) for 16 h. Mitomycin C (Mito, 10 µg/mL, 2 h) treatment served as a positive control for growth arrest. (**A**) Respective histograms of a representative donor. Pink line shows the sum the of the measurement. Blue represents the G0/G1 fraction, yellow the S phase, and green the G2/M fraction. (**B**) Following staining with PI: G0/G1 phase: for the control 45.6 ± 7.0%, ASC-CM 42.4 ± 16.6%, TGF-β1 61.5 ± 12.8%, and Mito C 65.3 ± 15.2%. S phase: control 42.3 ± 1.3%, ASC-CM 48.4 ± 14.9%, TGF-β1 30.2 ± 9.6%, and Mito 29.8 ± 14.7%. G2/M phase: control 10.9 ± 7.3%; ASC-CM 7.2 ± 2.9%; TGF-β1: 6.6 ± 3.5%, and Mito 5.3 ± 1.3%. The graph represents the mean percentages of cells. *n* = 3 independent biological donors. Statistical analysis: two-way ANOVA.

To assess whether KCs change their cell cycle status following distinct treatments, flow cytometric analysis was performed after PI staining (Figure 5). Cell cycle results indicated that TGF-β1 treatment led to an increased proportion of cells in the G0/G1 phase (61.5 ± 12.8%) compared to the control (45.6 ± 7.0%) and ASC-CM (42.4 ± 16.6%)-treated KCs. Further, TGF-β1 treatment resulted in decreased S phase (30.2 ± 9.6%) compared to the ASC-CM stimulation (48.4 ± 14.9%), and control (42.3 ± 1.3%). The G2/M phase did not differ between the treatments (control: 10.9 ± 7.3%; ASC-CM: 7.2 ± 2.9%; and TGF-β1: 6.6 ± 3.5%). Mitomycin C (Mito) served as a positive control for growth arrest [19]. The KCs treated with mitomycin C resulted in similar values as TGF-β1-treated KCs (G0/G1: 65.3 ± 15.2%, S phase: 29.8 ± 14.7%, and G2/M: 5.3 ± 1.3%), indicating that TGF-β1 induces growth arrest in the KCs (Figure 5).

### 2.6. Soluble Factors Secreted by the ASCs

**Figure 6:**
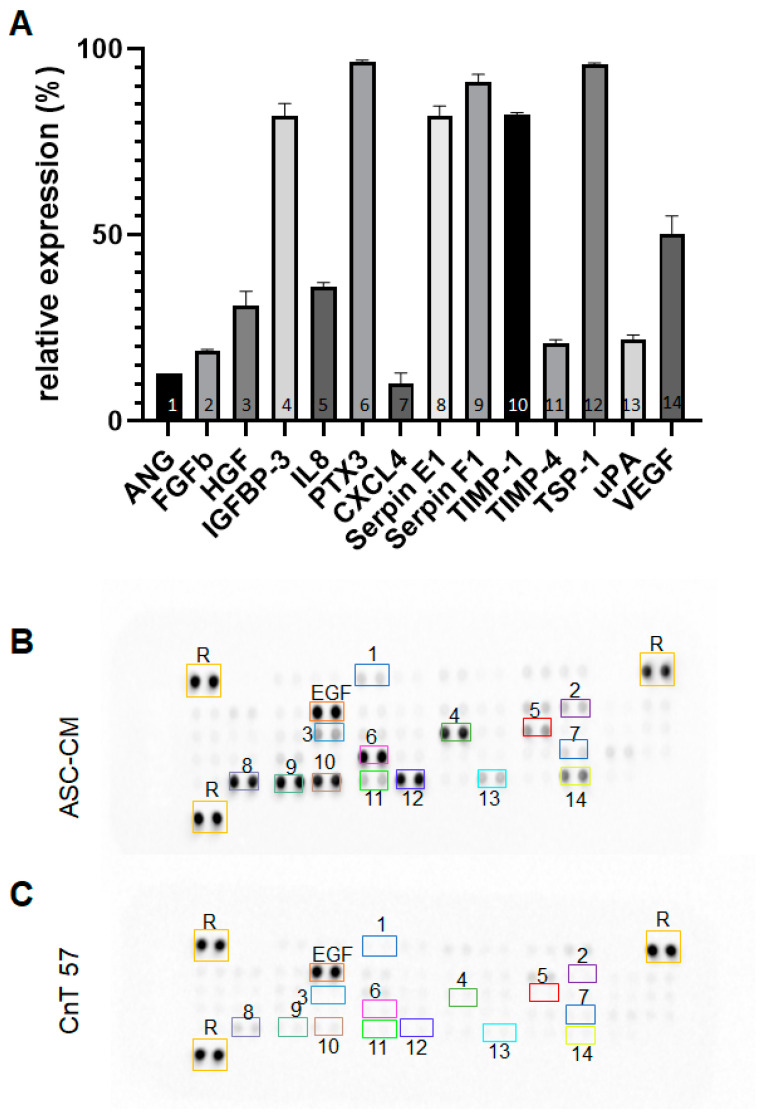
Protein array analysis of the ASC-CM. ASCs were cultured for 72 h in CnT57 medium. (**A**) Relative expression of detected proteins in the ASC-CM: ANG 12.9 ± 0%, FGFb 19.0 ± 0.2%, HGF 30.9 ± 2.7%, IGFBP-3 81.9 ± 2.4%, IL8 36.1 ± 0.8%, pentraxin 3 (PTX3) 96.7 ± 0.2%, CXCL4 10.2 ± 1.9%, Serpin E1 82.2 ± 1.8%, Serpin F1 92.2 ± 1.4%, TIMP-1 82.4 ± 0.4%, TIMP-4 20.8 ± 0.8%, TSP-1 95.7 ± 0.4%, uPA 21.8 ± 1%, and VEGF 50.4 ± 3.3%. Densitometry analysis relative to reference points (R). (**B**) Membrane array incubated with ASC-CM. (**C**) Negative control membrane incubated with CnT57 media only. Colored boxes in B and C show the relative expression of 1-14 cytokines detected using a protein array. EGF: epidermal growth factor. *n* = 5 independent donors.

We assessed the secretion of ASC-derived molecules using a protein array (Figure 6). We have detected the following 14 different proteins and polypeptides in the ASC-CM (*n* = 5 donors) and not in the control medium: basic fibroblast growth factor (FGFb), hepatocyte growth factor (HGF), insulin-like growth factor-binding protein (IGFBP-3), pentraxin 3 (PTX 3), platelet factor 4 (CXCL4), Serpins E1 and F1, tissue inhibitor of metalloproteinases (TIMPs 1 and 4), and thrombospondin-1 (TSP-1). ASCs also secreted immunomodulatory cytokines, including IL8, and the enzyme urokinase-type-plasminogen activator (uPA). Moreover, we detected pro-angiogenic factors, like vascular endothelial growth factor (VEGF) and angiogenin (ANG), in the ASC-CM.

### 2.7. Expression of KC Differentiation Markers Following ASC-CM and TGF-β1 Treatment

**Figure 7:**
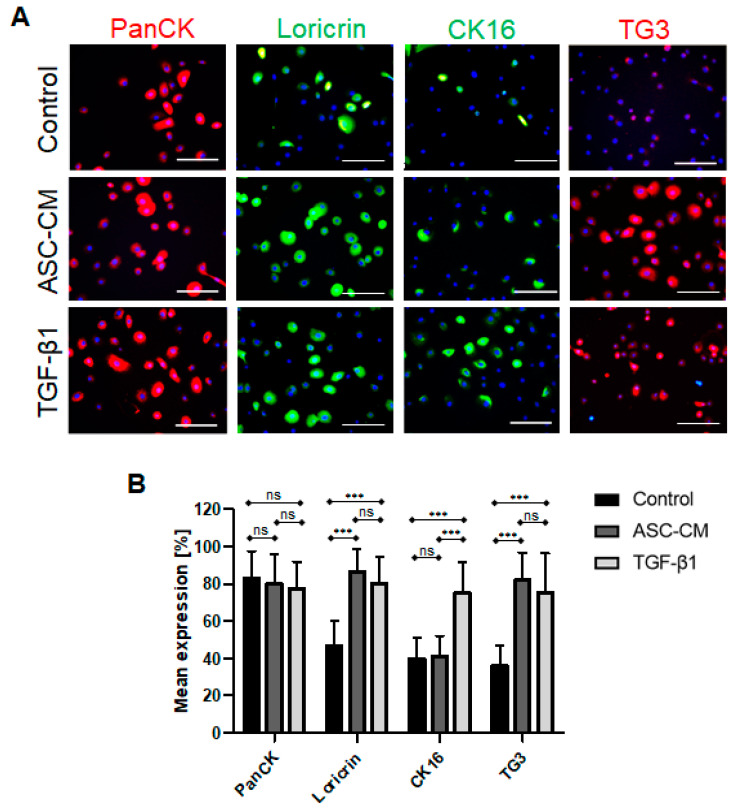
Protein expression of pan-CK, loricrin, CK16, and TG3 in untreated, ASC-CM-, or TGF-β1-treated KCs. (**A**) Immunofluorescence staining of KCs with pan-CK (red), loricrin (green), CK16 (green), and TG3 (red) antibodies. Images show increased loricrin and TG3 expression in TGF-β1- and ASC-CM-treated cells compared to untreated cells. Increased CK16 expression was detected in ASC-CM-treated cells compared to the TGF-β1-treated and untreated cells. (**B**) Quantification of positively stained cells. The graphs represent the mean ± SD percentage of cells positive for pan-CK, loricrin, CK16, and TG3 protein expression. The expression of pan-CK was similar regardless of treatment (ns, not significant), whereas loricrin and TG3 expression was enhanced in ASC-CM- and TGF-β1-treated cells (*** *p* < 0.001). In addition, an increased expression of CK16 (*** *p* < 0.001) was detected in TGF-β1-treated cells compared to the untreated and ASC-CM-treated cells. Scale bars = 30 μm. *n* = 6 independent donors.

The expression of specific differentiation KC markers was visualized using immunofluorescence staining (Figure 7). Pan-CK was used as a pan-marker of KCs (Figure 7A, first row). Accordingly, the quantification of the immunofluorescence staining of the pan-CK-positive KCs revealed a similar expression in all groups investigated: 83.1 ± 14.4% of total cells in the control, 80.3 ± 15.9% in the ASC-CM (*p* > 0.05 vs. control, ns), and 77.4 ± 14.4% in the TGF-β1-treated KCs (*p* > 0.05 vs. control, ns, *n* = 6) (Figure 7B).

Loricrin is a major component of the *stratum corneum*, which makes up 85% of a fully differentiated KC [2]. ASC-CM- and TGF-β1-treated KCs showed a significant higher expression of loricrin when compared to untreated KCs (Figure 7A). Accordingly, the quantification of the loricrin-positive KCs revealed 46.7 ± 13.7% of total cells in control, 86.7 ± 12.1% in ASC-CM (*p* < 0.001 vs. control), and 80.3 ± 14.3% in TGF-β1-treated KCs (*p* < 0.001 vs. control) (*n* = 6) (Figure 7B). The differences in the loricrin expression level between KCs treated with ASC-CM and TGF-β1 were not statistically significant (*p* < 0.05, ns).

CK16 is a wound healing marker induced in KCs in a hyperproliferative state or during differentiation to support wound closure [20]. In this study, KCs treated with ASC-CM showed an enhanced expression of CK16, while TGF-β1-treated and untreated KCs both demonstrated a lower number of CK16^+^ KCs (Figure 7A,B). The quantification of the immunofluorescence staining revealed 39.8 ± 11.2% of the CK16-positive KCs in the control, 41.2 ± 10.8% in the ASC-CM (*p* < 0.05 vs. control, ns), and 74.9 ± 16.8% in the TGF-β1 (*p* < 0.001 vs. control)-treated KCs (*n* = 6). The differences in the CK16 expression level between the ASC-CM and TGF-β1-treated KCs were statistically significant (*p* < 0.001).

Further, we sought to assess the expression of TG3, which is one of the pivotal enzymes responsible for the formation of protein polymers in the epidermis and the hair follicle [21]. As depicted in Figure 7B, we confirmed that TG3 was expressed at a higher level in the treated cells as compared to the control. The quantification of the immunofluorescence staining of the TG3-positive KCs revealed 35.9 ± 10.8% expression in control, 82.0 ± 14.8% (*p* < 0.001 vs. control) in ASC-CM, and 75.4 ± 20.9% (*p* < 0.001 vs. control) in TGF-β1-treated KCs (*n* = 6) (Figure 7B). The differences in the TG3 expression level between KCs treated with ASC-CM and TGF-β1 were not statistically significant (*p* < 0.05, ns).

### 2.8. Distinct Expression Pattern of Suprabasal and Basal Epidermal Markers in KCs Stimulated with ASC-CM or TGF-β1 

**Figure 8:**
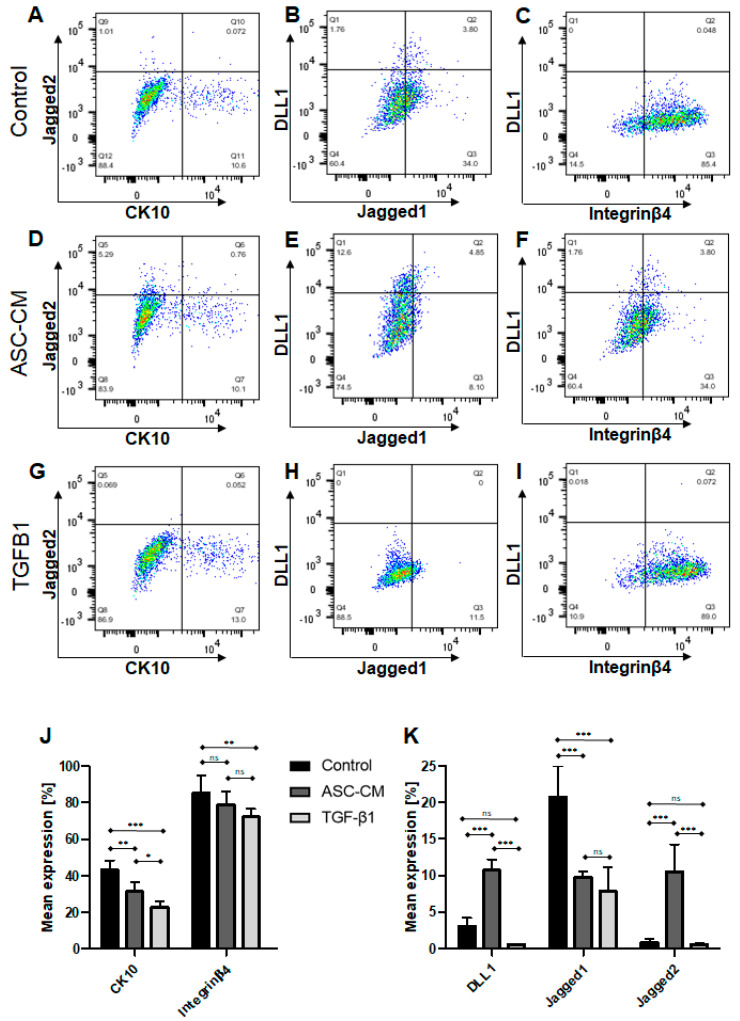
Protein expression of different suprabasal and basal epidermal markers. (**A**–**C**) Representative dot blots of untreated KCs (**A**–**C**), ASC-CM-treated KCs (**D**–**G**), or TGF-β1-treated KCs (**G**–**I**). (**A**,**D**,**G**) Jagged1 and CK10 co-expression. (**B**,**E**,**H**) DLL1 and Jagged1 co-expression. (**C**,**F**,**I**) DLL1 and Integrinβ4 co-expression. (**J**–**K**) Quantification of the mean % of expression ± SD. The graph in (**J**) shows decreased CK10 expression in ASC-CM- and TGF-β1-treated KCs (** *p* < 0.01 and *** *p* < 0.001, respectively, ASC-CM compared to TGF- β1 * *p* < 0.05) and decreased Integrinβ4 in TGF-β1-treated KCs (** *p* < 0.01). The graph in (**K**) shows that DLL1 and Jagged2 expression was increased in ASC-CM-treated KCs (*** *p* < 0.001) compared to untreated and TGF-β1-challenged KCs, whereas Jagged1 was higher in untreated KCs and downregulated in both ASC-CM- and TGF-β1-stimulated KCs (*** *p* < 0.001). *n* = 5 independent donors.

Cytokeratin 10 (CK10) is considered a late epidermal differentiation marker, while Integrinβ4 is prevalent in the KC basal layer of the epidermis [22]. Flow cytometric analyses demonstrated that the expression of CK10 was reduced in KCs following both treatments with ASC-CM or TGF-β1 (Figure 8). In contrast, Integrinβ4 was expressed in KCs at a similar level before and after treatment with ASC-CM, but was lower in TGF-β1-treated cells (Figure 8). Accordingly, the quantification of the flow cytometric analyses of the CK10-positive KCs revealed 43.0 ± 5.21% positive cells in control, 31.2 ± 5.4% in ASC-CM (*p* < 0.01 vs. control), and 22.5 ± 3.3% in TGF-β1 (*p* < 0.001 vs. control)-treated KCs. The analysis of Integrinβ4-positive cells revealed 85.3 ± 9.4% in control, 79.0 ± 6.9% in ASC-CM, and 72.2 ± 4.2% in TGF-β1 (*p* < 0.01 vs. control) treated KCs (*n* = 5) (Figure 8B).

Further, to investigate whether the TGF-β1 effects on proliferation and differentiation of KC are linked with the Notch signaling pathway, the expression of three Notch ligands, namely Jagged1 (suprabasal expression in the KCs of the epidermis), Jagged2, and DLL1 (basal expression in the KCs of the epidermis), were analyzed using flow cytometry (Figure 8).

We detected that the expression of the basal epidermal markers DLL1 and Jagged 2 was specifically increased upon treatment with the ASC-CM. The quantification of the DLL1-positive KCs revealed 3 ± 1.15% in control, 10.67 ± 1.5% in ASC-CM (*p* < 0.001 vs. control), and 0.5 ± 0.12% in TGF-β1 (*p* > 0.05 vs. control, ns)-treated KCs.

Similar, the expression of Jagged2 showed 0.8 ± 0.5% in control, 10.5 ± 3.8% in ASC-CM (*p* < 0.001 vs. control), and 0.5 ± 0.2% in TGF-β1 (*p* > 0.05 vs. control, ns)-treated KCs. Thus, both DLL1 and Jagged2 were significantly increased in ASC-CM-treated KCs compared to untreated and TGF-β1-treated KCs (*p* < 0.001).

Further, Jagged1, a suprabasal epidermal Notch ligand, showed the highest expression from all three ligands, especially in untreated (control) KCs, and decreased expression following the ASC-CM and TGF-β1 treatments. Furthermore, Jagged1 quantification revealed 20.8 ± 4.2% positive KC in control, 9.7 ± 0.8% in ASC-CM (*p* < 0.001 vs. control), and 7.8 ± 3.4% in TGF-β1 (*p* < 0.001 vs. control) treated cells. Thus, the expression of Jagged 1 was similar between the ASC-CM and TGF-β1 treatments (*p* > 0.05, ns).

## 3. Discussion

In this study, by treating human primary KCs with ASC-CM and/or synthetic TGF-β1, we observed (1) significant differences in KC proliferation between the ASC-CM and TGF-β1 treatments, with reduced KC numbers upon the latter one, (2) enhanced migration and wound closure of the KCs upon ASC-CM treatment, (3) increased proportion of cells in the S phase in ASC-CM-treated KCs as compared to the TGF-β1 treatment, which promoted sharp cell cycle arrest with the increased proportion of cells in the G0/G1 phase, (4) reduced expression of CK10 triggered by both conditions, (5) enhanced expression of TG3 and loricrin induced by both treatments, and (6) increased DLL1 and Jagged2 basal KC marker expression under the ASC-CM condition, whereas suprabasal Jagged1 was decreased in both ASC-CM- and TGF-β1-stimulated KCs.

As shown in our study, TGF-β1 inhibited the proliferation and migration of KCs. In general, these results are in line with the previously published study of Matsumoto *et al.* [23]. In contrast, ASC-CM-treated KCs showed an enhanced proliferation and migration rate in our study. In particular we showed that the ASC-CM enhanced the closure of scratch wounds in primary skin KC monolayers already after 20 h, whereas wounds created by the TGF-β1-treated KCs were still open after 72 h, with the KCs undergoing morphological changes into roundish, single cells, indicating their terminal differentiation [24].

These effects are consistent with results reported for the rate of cutaneous skin wound closure in vivo in mice with impaired responsiveness to TGF-β1, suggesting that the in vivo endogenous activation of TGF-β1 might actually suppress the rate of wound closure [25]. These mice have dramatically accelerated wound closure, as expected, since endogenously activated TGF-β1 normally slows the rate of wound closure.

Moreover, the study concluded that TGF-β1 signaling in vivo in keratinocytes ultimately leads to the inhibition of their functions crucial to wound repair, namely KC proliferation and migration, and finally delayed wound re-epithelialization in a Smad3-dependent manner [25].

Further, we analyzed the cell cycle of treated KCs in order to verify that the reduced proliferation of TGF-β1-treated KCs was a result of growth arrest. Indeed, we found that KCs challenged with TGF-β1 demonstrated an arrest in the G0/G1 phase, similar to the mitomycin C-treated KCs. Mitomycin C is a known inhibitor of proliferation [26]. It was shown that treatment with mitomycin C of different cells induced a shift towards the G0/G1 phase [19,27]. Therefore, the reduced proliferative and migrative capacity of TGF-β1-stimulated KCs is most likely induced by promoting cell cycle arrest in G0/G1. However, the ASC-CM treatment increased DNA synthesis (S phase), leading to cell cycle progression and, therefore, also proliferation and migration of the cells, which is essential in terms of wound healing.

We hypothesized that this effect is closely related to the ASC secretome and the secreted soluble factors, that are known to enhance the proliferation and migration of KCs [28]. Indeed, several growth factors, such as HGF, FGFb, and VEGF, have been identified within the ASC secretome in this study. Interestingly, these factors were proved to have the strongest direct mitogenic effect on KCs in experimental systems [29]. Furthermore, HGF and its receptor c-Met were shown to promote the proliferation of KCs in culture by inducing the dissociation of KC sheets, leading to higher numbers of individual, scattered KCs that was accompanied by an increase in their motility [30,31]. Moreover, VEGF was shown to be a potent stimulus of KC proliferation and migration in vitro and in vivo in mice by interacting with VEGF-R1/2 on the KCs [32]. Another factor involved in wound healing is IL8, since it increases the rate of cell migration in KCs [33]. 

In addition, Shen *et al.* demonstrated that uPA plays a central role in KC migration, and its ablation leads to delayed wound healing, abundant neutrophil accumulation, and persistent fibrin deposition in uPA-deficient mice [34]. Interestingly, its counterpart, Serpin E1, also known as PAI-1, was highly expressed in the ASC secretome in our study. Furthermore, Serpin E1 was shown to be essential for optimal KC monolayer wound repair [35]. Therefore, the ASC secretome represents a unique composition, as it not only contains factors that induce proliferation, but also inhibitory factors, maintaining the homeostasis, also shown, for example, by the presence of insulin-like growth factor-binding protein-3 (IGFBP-3) [36].

However, we could not detect TGF-β1 in the secretome, since the cytokine array only detects LAP, which is the proprotein of TGF-β1. However, in our previous study, we demonstrated an enhanced expression and secretion of TGF-β1 in ASCs as compared to dermal fibroblasts [14].

Further, we detected several specific differences in the expression patterns of KC markers. In particular, we observed that TGF-β1 up-regulated CK16 expression in KC in vitro. CK16 is used as an epithelial wound healing marker of activated KCs, which are proliferating and migrating during active healing process [1]. Indeed, previous reports demonstrated enhanced CK16 expression by KCs near the wound site upon injury, where the highest expression of TGF-β1 was also reported [37].

Along with the increase in CK16 expression in KCs following TGF-β1 treatment, a decrease in CK10 was observed in our study. Interestingly, TGF-β1 significantly stimulated CK10 protein downregulation in KCs as compared to the ASC-CM-treated cells. Therefore, we hypothesized that the presence of TGF-β1 might mimic the presence of a wound situation in vitro, since TGF-β1 is particularly secreted by platelets upon injury [38]. However, CK10 is also a marker of suprabasal differentiated KCs [37], suggesting that TGF-β1 downregulates KC differentiation in vitro. Moreover, under the influence of hyperproliferative stimuli, for example during wound healing and in certain disorders including cancer, epidermal expression levels of CK1 and CK10 was reported to be drastically reduced [38]. Those findings were further supported by Li and his colleagues, who demonstrated that TGF-β1, a potent keratinocyte growth inhibitor, has been shown to be overexpressed in keratinocytes in certain inflammatory skin diseases, thus counteracting the effects of other growth factors at the site of inflammation [39].

Interestingly, the effect of TGF-β1 on cultured KC differentiation also depends on the calcium ions (Ca^2+)^ in the culture medium. Matsumoto *et al*. found that TGF-β1 enhances KC differentiation under high Ca^2+^ environments, while inhibiting it under low Ca^2+^ conditions [23]. Recently, another group also reported similar results [3]. These findings are in accordance with our results regarding low CK10 expression by KCs upon TGF-β1 stimulation in vitro since we used a Ca^2+^-free KC medium in our study.

Further, in this study, we observed an enhanced expression of loricrin and transglutaminase 3 (TG3) in KCs treated with ASC-CM and TGF-β1 as compared to control cells, indicating a pro-differentiation effect of the ASC-CM and TGF-β1 on KCs. Since transglutaminases support the cross-linking of keratins [21], this indicates that the treated cells possessed improved inter-protein bond formation, a feature observed in the final step of epidermal KC differentiation [1]. Further, it has been previously demonstrated that transglutaminases increase TGF-β1 mRNA and protein expression via a nuclear transcription factor (NF)-κB signaling mechanism [40]. Importantly, the signaling activation results in a positive feedback loop, in which TGF-β1 and transglutaminases display reciprocal activation of expression [41]. These data confirm our results, since we demonstrated that ASC-CM-treated cells showed the highest expression of TG3, suggesting that ASCs might secrete other specific factors which further enhance the expression of TG3 [42].

The TGF-β1 isoform is known to mostly subside in the upper layers of the epidermis, such as the *stratum corneum* and *stratum granulosum* [6], which could explain why this growth factor improves the expression of cross-linking proteins within the KC cultures. To support this hypothesis, Hata *et al*. previously reported that predominantly suprabasal KCs of the epidermis secrete a latent form of TGF-β1, which can be activated through different stimuli and exert their specific functions [43]. Taken together the enhanced expression of loricrin and TG3 suggests that similar to physiological conditions in normal skin, TGF-β1 induces the terminal differentiation of KCs in vitro.

In the skin, the distribution of integrins is restricted to the basal keratinocytes [44]. In particular, Integrinβ4 is a KC marker specifically expressed in the basal layer, where it mediates the adhesion of KCs to the basement membrane [45]. As the initiation of the maturation in skin is associated with the detachment of cells from the basement membrane, the downregulation of Integrinβ4 normally occurs during the process of terminal KC differentiation. Our data showed a significant reduction in Integrinβ4 protein levels in cells stimulated with either ASC-CM or TGF-β1. These findings confirm previous reports on the terminal differentiation of KCs, where integrin expression is downregulated when the KCs start the differentiation process [44].

Further, in this study, the expression of basal epidermal markers, such as the Notch ligands DLL1 and Jagged2, showed rather low levels in cultured KCs; however, the ASC-CM treatment specifically increased the expression of these basal epidermal Notch ligands in the KCs in vitro. In contrast, treatment with TGF-β1 led to a significant decrease in the DLL1 and Jagged2 ligands, as well as Jagged1, which is a marker of suprabasal KCs in the epidermis. 

Thus, we have shown that the application of the ASC-CM specifically increases the numbers of basal KCs in vitro, assumable by their increased adhesion and proliferation. Therefore, we suggest that ASC-CM treatment applied during the expansion of KCs could be used for therapeutic application to obtain high numbers of potent basal KCs in a shorter time. We hypothesize that ASCs secrete a specific cocktail of pro-regenerative cytokines that modulate KC growth and migration in vitro [42]. Importantly, our findings are of crucial importance for the tissue engineering of skin transplants, and in general for KC-based stem cell therapies, since epidermal basal layer-derived KCs show the highest adhesion and proliferation rates [46].

Moreover, increasing attention is focusing on the use of the ASC-CM to treat chronic non-healing wounds that involve a pathologically prolonged inflammatory state [47,48,49]. In this scenario, the ASC-CM can be used as a new cell-free therapy with biological activity similar to the ASCs, thus representing another promising novel therapy to treat chronic wounds through the trophic, paracrine, and immunomodulatory properties of the ASC secretome [28]. Indeed, several in vivo experiments support a positive effect of the ASC-CM in different in vivo skin wound healing models; for example, Park *et al*. observed accelerated wound closure following a topical application of the ASC-CM on full-thickness excisional skin wounds [50]. Furthermore, Irons *et al*. recently showed enhanced diabetic wound healing after topical application of the ASC-CM in Yorkshire pigs, in particular, due to increased cell proliferation and immunomodulation effects [51]. Further, using the rat skin excisional wound healing model, Su *et al.* subcutaneously injected the ASC-CM into skin lesions and to the bed of the wound [52]. This group demonstrated beneficial therapeutic effects in the wounds treated with the ASC-CM.

To conclude, we observed in this study that the sole TGF-β1 treatment reduces proliferation and migration. Furthermore, it seems to drive KCs into the terminal differentiation state by reducing the expression of CK10 and the basal epidermal markers, such as the DLL1 and Jagged2 Notch ligands. Furthermore, TGF-β1-treated KCs increased the expression of loricrin and TG3, which are late differentiation markers. In contrast, ASC-CM-treated KCs showed other expression patterns, confirming that ASCs also secrete pro-regenerative cytokines which appear to maintain the KCs in a proliferative state, with increased expression of the epidermal basal markers DLL1 and Jagged2.

However, the effects observed in this study were detected and measured in KCs following 2D in vitro cultivation that represents a possible limitation of this study, since it is known that KC interacts closely with dermal fibroblasts in the skin. The lack of those cell–cell contacts might modulate the expression of certain differentiation markers [14,53,54]. Therefore, ideally, a 3D culture system should be applied in future investigations to understand the interplay between ASC-CM and/or TGF-β1 and the KCs.

Notably, our data demonstrate that the ASC-CM enhances the functions of keratinocytes in a paracrine fashion. Therefore, we believe that the stimulatory effect of the ASC-CM on cutaneous wound healing may be partially mediated by the paracrine effects of the ASC-CM on keratinocytes, as well as other skin cells. Hence, application of the ASC-CM could be an innovative therapeutic approach in the treatment of large, severe skin defects, chronic wounds, and other conditions.

## 4. Materials and Methods

### 4.1. Human Skin and Adipose Samples

All experiments were performed according to the “Declaration of the Helsinki Principles”. Human foreskin (infant) and adipose (adults) samples were acquired from patients after approval was obtained from the Ethics Committee of the Canton Zurich (BASEC-Request-Nr. 2018-00269), and informed consent was given by parents (infant foreskin) and patients (adipose sample).

The human subcutaneous adipose tissue samples were obtained from healthy human donors between the ages of 18 and 68 years. The samples were obtained from surgical fat liposuction, mostly from the abdominal, arm, or leg body areas (BASEC-Request-Nr. 2018-00269).

### 4.2. Isolation and Culture of Human Primary Cells

Keratinocytes (KC) (*n* = 5 independent donors) were isolated from infant foreskin. The epidermis was separated from the dermis and digested with 0.5% 10× concentrated trypsin/EDTA (Gibco, Fisher Scientific, Reinach, Switzerland) for 2 min (minutes) at 37 °C under shaking. The mixture was centrifuged at 200× *g* for 5 min and the supernatant was discarded. The cells were then resuspended in CnT57.S medium (CELLnTEC advanced cell systems AG, Bern, Switzerland) with 1% penicillin/streptomycin (PenStrep) (100x concentrated, Gibco, Fisher Scientific, Reinach, Switzerland) and no serum before being counted. Afterwards, approximately 10 × 10^4^ cells were added to each 5 cm^2^ plate, which had been previously coated with 1:50 type 1 acido-soluble collagen (8 mg/mL, Symatese, Chaponost, France) diluted in Dulbecco’s phosphate-buffered saline (DPBS, Sigma-Aldrich Chemie GmbH, Buchs, Switzerland).

ASCs were isolated from the stromal vascular fraction (SVF) of human white adipose tissue. For this, subcutaneous fat samples were minced into small pieces and digested with 0.075% (*W*/*V*) type II collagenase (355 U/mg, Worthington, Lakewood, NJ, USA) for 1 h at 37 °C under shaking. The mixture was centrifuged at 200× *g* for 10 min; afterwards, the oil and aqueous layers were discarded.

The resulting pellet was washed in DPBS (Sigma-Aldrich Chemie GmbH, Buchs, Switzerland) and passed first through a 100 µm strainer, followed by a 40 µm strainer (Sigma-Aldrich Chemie GmbH, Buchs, Switzerland) to get rid of debris. Red blood cells were lysed through incubation for 2 min with a buffer containing 0.15 M ammonium chloride, 1.0 mM potassium bicarbonate (both Merck, Darmstadt, Germany), and 0.1 mM Na-EDTA (Fluka Analytical, Sigma-Aldrich Chemie GmbH, Buchs, Switzerland). After centrifugation and washing in DPBS, the SVF cell pellet was resuspended in complete α-Modified Eagle’s medium (α-MEM, Gibco, Fisher Scientific, Reinach, Switzerland) supplemented with 10% fetal bovine serum (FBS), 50 ng/mL FGF-2 (Peprotech, Hamburg, Germany), 1% HEPES, 1% sodium pyruvate, and 1% penicillin–streptomycin–glutamine (100×) solution (all obtained from Gibco, Fisher Scientific, Reinach, Switzerland). The cells were incubated at 37 °C and 5% CO_2_.

### 4.3. Preparation of the ASC-CM

ASCs (4.3 × 10^6^ cells, *n* = 5 different donors) were inoculated in 75 cm^2^ flasks containing complete α-MEM medium, as seen above, and incubated overnight at 37 °C at 5% CO_2_. The following day, the medium was exchanged for serum-free CnT57.S medium (CellnTec, Bern, Switzerland) with 1% PenStrep (100× concentrated, Gibco, Fisher Scientific, Reinach, Switzerland) and the cultures were incubated for another 72 h. After 72 h of incubation, the conditioned medium (ASC-CM) was collected, centrifuged at 340× *g* for 5 min, filtered through a 0.22 μm syringe filter (Sigma-Aldrich Chemie GmbH, Buchs, Switzerland), and stored at −80 °C for future use.

### 4.4. Treatment of Human KCs with the ASC-CM or Synthetic TGF-β1

Human primary KCs were cultured in the ASC-CM or in the serum-free CnT57.S medium (CellnTec, Bern, Switzerland) supplemented with TGF-β1 (2.5 ng/mL, Peprotech, Hamburg, Germany) or in CnT57.S medium as control (CellnTec, Bern, Switzerland) for 72 h.

### 4.5. Differentiation Assays

For adipogenic, osteogenic, and chondrogenic differentiation, adipose-derived stromal cells (ASCs) (P3, *n* =3 independent donors) were seeded onto 5 cm cell culture dishes and cultivated in complete α-MEM medium with the addition of recombinant human FGF (50 ng/mL, PeproTech, Hamburg, Germany) until they reached the confluence of 80–90%. Subsequently, the regular culture medium was replaced with respective differentiation media for an additional 2–4 weeks. For adipogenic differentiation, ASCs were cultivated in MesenCultTM adipogenic differentiation medium (StemCell, Cologne, Germany), according to the supplier instructions. For osteogenic differentiation, the cells were cultured with low glucose DMEM (Thermo Fisher Scientific, Basel, Switzerland) supplemented with 0.1 μM dexamethasone, 10 mM glycerol phosphate, 50 μM L-ascorbic acid, 10% FBS, and 50 μg/mL gentamycin (all from Thermo Fisher Scientific, Basel, Switzerland). The chondrogenic differentiation medium consisted of high glucose DMEM (Thermo Fisher Scientific, Basel, Switzerland) supplemented with 50 μg/mL L-ascorbic acid, 40 μg/mL L-proline, 1% ITS™+ Premix (5 μg/mL insulin, 5 μg/mL transferrin, and 5 ng/mL selenious acid), 10 ng/mL TGF-β3, and 50 μg/mL gentamycin.

### 4.6. Oil Red O Staining

Oil Red O staining was performed to detect adipogenic differentiation. The differentiated ASCs or undifferentiated ASCs (control) were fixed in 4% PFA for 15 min at RT. Subsequently, cells were washed with isopropanol and stained with Oil Red O solution (Sigma-Aldrich GmbH, Buchs, Switzerland) for 15 min at room temperature (RT). After washing in deionized water, the cells were visualized and pictures were taken with a DXM1200F digital camera connected to a Nikon Eclipse TE2000-U inverted microscope (Nikon Europe B.V., Egg, Switzerland). Images were processed with Photoshop 7.0 (Adobe Systems Inc., Munchen, Germany).

### 4.7. Alizarin Red Staining

Alizarin red staining was performed to detect osteogenic differentiation, as previously described by Park *et al*. [55]. The differentiated ASCs (P3) or undifferentiated ASCs (control) (P3) were fixed with 4% PFA for 15 min at RT, and stained with 2% alizarin red S (Sigma-Aldrich GmbH, Buchs, Switzerland) in ddH2O solution for 30 min at RT. After washing in deionized water, the cells were visualized and pictures were taken with a DXM1200F digital camera connected to a Nikon Eclipse TE2000-U inverted microscope (Nikon Europe B.V., Egg, Switzerland). Images were processed with Photoshop 7.0 (Adobe Systems Inc., Germany).

### 4.8. Alcian Blue Staining

Alcian blue staining was used to detect chondrogenic differentiation, as previously described by Yu *et al*. [56]. The differentiated ASCs (P3) or undifferentiated ASCs (control) (P3) were fixed in 4% PFA for 15 min at RT and stained with 1% alcian blue in glacial acetic acid solution (Alfa Aesar, Thermo Fisher Scientific, Basel, Switzerland) for 30 min. After washing in deionized water, the cells were visualized and pictures were taken with a DXM1200F digital camera connected to a Nikon Eclipse TE2000-U inverted microscope (Nikon Europe B.V., Egg, Switzerland). Images were processed with Photoshop 7.0 (Adobe Systems Inc., Germany).

### 4.9. Fluorescein Diacetate (FdA)/Propidium Iodide (PI) Staining

For the live/dead staining, untreated, ASC-CM- and TGF-β1-treated keratinocytes (*n* = 3 independent donors) were concomitantly stained with FdA (80 μg/mL; Sigma-Aldrich GmbH, Buchs, Switzerland) and PI (200 μg/mL; Sigma-Aldrich GmbH P4864, Buchs, Switzerland). The viability assay was performed after 72 h of culture. Cells were visualized and pictures were taken with a DXM1200F digital camera connected to a Nikon Eclipse TE2000-U inverted microscope (Nikon Europe B.V., Egg, Switzerland). Images were processed with Photoshop 7.0 (Adobe Systems Inc., Germany).

### 4.10. Wound Healing Assay

Keratinocytes (*n* = 3 independent donors) were isolated, as described above, and seeded into a 24-well plate in CnT57.S medium (10 × 10^4^ cells/well). Plates were incubated at 37 °C and 5% CO_2_ until a nice monolayer was formed (3–4 days). Once the monolayer was formed, a scratch was made using a 200 µL tip in the vertical direction. After the scratch, the wells were washed once with PBS. Keratinocytes were treated as described above for 72 h. Time-lapse imaging were performed using a Nikon ECLIPSE Ti2 inverted microscope (Nikon Europe B.V., Egg, Switzerland) in order to take pictures every 30 min at three different points along the scratch per well. The scratch area was analyzed using ImageJ/FIJI software (ver. 1.53i, NIH, Bethesda, MA, USA).

### 4.11. Cell Cycle Analysis

Keratinocytes (*n* = 3 independent donors) were isolated as described above and seeded into a 6-well plate in CnT57.S medium (20 × 10^4^ cells/well). Once they reached a confluency of 80%, they were treated as described above. After 16 h, the cells were detached using 2x trypsin/EDTA, following which the cell suspension was collected and centrifuged for 3 min at 350× *g*, and the cell pellet was fixed using 70% EtOH. As a positive control, KCs were treated with mitomycin C (10 µg/mL) for 2 h and also collected and fixed as described above. For fixation, EtOH was added dropwise while vortexing. Samples were kept at −20 °C. On the day of FACS, cells were washed 2x with PBS and then stained with 500 µL of the PI-RNase Mix. The PI-RNase Mix was prepared shortly before staining the cells, consisting of 100 µg/mL RNase and 100 µg/mL PI in PBS. The cells were incubated at RT for 40 min. After incubation, 3 mL of FACS buffer was added and centrifuged for 3 min at 350× *g*. The cell pellet was resuspended in 300 µL of FACS buffer and analyzed with a LSR Fortessa flow cytometer (BD Biosciences, London, UK, provided by the Center for Microscopy and Image Analysis, University of Zurich, Zurich, Switzerland). The analysis of flow cytometry data was performed using FlowJo^TM^.

### 4.12. Cytokine Array

The secretome of ASCs (*n* = 5 independent donors) was measured using a human angiogenesis array obtained from R&D systems. For this, 500 µL of the ASC-CM was incubated on the membrane, according to the manufacturer’s instruction. Analysis was performed through the densitometric method using ImageJ/FIJI software (ver. 1.53i, NIH, Bethesda, MA, USA). Expression was calculated relative to the reference points following the background subtraction of the medium alone.

### 4.13. Flow Cytometry 

ASCs (*n* = 3 independent donors) from the isolated SVF fraction were detached using 0.5% 10× concentrated trypsin/EDTA. The cell suspension was collected and centrifuged for 3 min at 350× *g*, following which the cell pellet was resuspended in 100 µL of FACS buffer (composed of 0.5% human serum albumin and 0.5 mM EDTA in DPBS) and incubated with the following conjugated antibodies (directly diluted in FACS buffer, dilution 1:20): PE-conjugated mouse anti-human CD44 monoclonal antibody (Clone G44-26, BD Biosciences, Allschwil, Switzerland), PE-conjugated mouse anti-human CD73 monoclonal antibody (Clone AD2, BD PharmingenTM, BD Biosciences, Allschwil, Switzerland), AlexaFluor 647-conjugated CD90 monoclonal antibody (clone: 5E10, BioLegend, Lucerna-Chem AG, Lucerne, Switzerland), FITC-conjugated mouse anti human CD105 monoclonal antibody (Clone 43A3, BioLegend, Lucerna-Chem AG, Lucerne, Switzerland), FITC-conjugated mouse anti-human CD14 monoclonal antibody (Clone M5E2, BD PharmingenTM, BD Biosciences, Allschwil, Switzerland), and PE-conjugated mouse anti-human CD31 (clone: WM59. BioLegend, Lucerna-Chem AG, Lucerne, Switzerland). The following isotype IgGs were used as isotype controls (directly diluted in FACS buffer, dilution 1:20): AlexaFluor 647-conjugated mouse isotype control (clone MOPC-21, BioLegend, Lucerna-Chem AG, Lucerne, Switzerland), PE-conjugated isotype control (clone MOPC-21, BioLegend, Lucerna-Chem AG, Lucerne, Switzerland), and FITC-conjugated isotype control (clone MOPC-21, BioLegend, Lucerna-Chem AG, Lucerne, Switzerland). Additionally, all samples, except for the unstained controls, were stained with live/dead Zombie aqua solution (BioLegend, London, UK, dilution 1:600 in 100 µL FACS Buffer) prior to fixation with 2% PFA. After washing, cells were resuspended in FACS buffer and analyzed with a LSR Fortessa flow cytometer flow cytometer (BD Biosciences, London, UK, provided by the Center for Microscopy and Image Analysis, University of Zurich, Zurich, Switzerland). The analysis of flow cytometry data was performed using density plots in FlowJoTM (BD, Biosciences, London, UK), where the background staining was determined using the isotype controls (Supplementary Figure S1).

Human primary KCs (*n* = 5 independent donors) at passage P2-3 were detached after treatment using 0.5% 10× concentrated trypsin/EDTA. The cell suspension was collected and centrifuged for 3 min at 353× *g*. The cell pellet was resuspended in 100 µL of FACS buffer (composed of 0.5% human serum albumin and 0.5 mM EDTA in DPBS) and incubated with the following conjugated antibodies (directly diluted in FACS buffer, dilution 1:20):

PE-conjugated mouse anti-human Jagged 2 monoclonal antibody (clone MHJ2-523, BioLegend, Lucerna-Chem AG, Lucerne, Switzerland), APC conjugated mouse anti-human CK10 monoclonal antibody (clone VIK-10, NovusBio, R&D Systems Europe Ltd., Abingdon, UK), PE-conjugated mouse anti-human DLL1 monoclonal antibody (clone MHD1-314, BioLegend, Lucerna-Chem AG, Lucerne, Switzerland), FITC-conjugated mouse anti-human Jagged 1 monoclonal antibody (clone E12, Santa Cruz Biotechnology, Labforce AG, Muttenz, Switzerland), and FITC-conjugated mouse anti-human Integrin-β4 monoclonal antibody (clone 422325, R&D Systems Europe Ltd., Abingdon, UK). The following isotype IgGs were used as isotype controls (dilution 1:20 in FACS buffer): APC-conjugated mouse isotype control (clone MOPC-21, BioLegend, Lucerna-Chem AG, Lucerne, Switzerland), PE-conjugated isotype control (clone MOPC-21, BioLegend, Lucerna-Chem AG, Lucerne, Switzerland), and FITC-conjugated isotype control (clone MOPC-21, BioLegend, Lucerna-Chem AG, Lucerne, Switzerland). Additionally, all samples, except for the unstained controls, were stained with live/dead Zombie aqua solution (BioLegend, dilution 1:600 in FACS Buffer) prior to fixation with 2% PFA. After washing, cells were resuspended in FACS buffer and analyzed with a LSR Fortessa flow cytometer flow cytometer (BD Biosciences, London, UK, provided by the Center for Microscopy and Image Analysis, University of Zurich, Zurich, Switzerland). The analysis of flow cytometry data was performed using density plots in FlowJoTM (BD, Biosciences, London, UK), where the background staining was determined using the isotype controls (Supplementary Figure S1).

### 4.14. Immunofluorescence Staining

Immunofluorescence staining was performed as described by Biedermann *et al*. [57]. Briefly, cells (*n* = 6 independent donors) cultured on type 1 collagen-coated plates (Symatese, Chaponost, France) were fixed and permeabilized in an acetone/methanol mixture for 5 min at −20 °C, air dried, and washed three times in DPBS (Sigma-Aldrich GmbH, Buchs, Switzerland), followed by blocking with 2% BSA in DPBS (Sigma-Aldrich GmbH, Buchs, Switzerland) for 30 min.

Incubation with the primary antibodies (see below in the text) was performed in blocking buffer (2% BSA/PBS) overnight at 4 °C. Afterwards, the plates were washed three times for 5 min in DPBS (Sigma-Aldrich GmbH, Buchs, Switzerland) and blocked for an additional 15 min before the secondary antibody (text below) was added and incubated for 1 h at room temperature. Finally, the plates were incubated for 5 min in DPBS (Sigma-Aldrich GmbH, Buchs, Switzerland) containing 1 μg/mL DAPI (Sigma-Aldrich GmbH, Buchs, Switzerland), washed twice for 5 min in DPBS (Sigma-Aldrich GmbH, Buchs, Switzerland), and mounted in Dako mounting solution (Dako, Baar, Switzerland). Pictures of immunofluorescence staining were taken with a DXM1200F digital camera connected to a Nikon Eclipse TE2000-U inverted microscope (Nikon Europe B.V., Egg, Switzerland). Images were processed with Photoshop 7.0 (Adobe Systems Inc., Germany).

The KCs were stained using the following primary antibodies: rabbit anti-human loricrin polyclonal antibody (clone ab85679, abcam, Cambridge, UK), mouse anti-human CK16 monoclonal antibody (clone ab80574, abcam, Cambridge, UK), and rabbit anti-human transglutaminase 3 polyclonal antibody (clone NBP1-86950, NovusBiologicals, R&D Systems Europe Ltd., Abingdon, UK). This step was then followed by incubation with the following secondary antibodies: FITC rabbit anti-mouse polyclonal antibody (catalog number F0313, Agilent Technologies Schweiz AG, Basel, Switzerland), FITC pig anti-rabbit polyclonal antibody (catalog number F0205, Agilent Technologies Schweiz AG, Basel, Switzerland), and TRITC goat anti-rabbit polyclonal antibody (catalog number sc-3841, Santa Cruz Biotechnology, Labforce AG, Muttenz, Switzerland).

Additionally, a mouse anti-human pan-CK monoclonal antibody (clone C11, Santa Cruz Biotechnology, Labforce AG, Muttenz, Switzerland) was labelled using the Zenon Alexa Fluor 555 Mouse IgG1 Labeling Kit (catalog number Z25005, Invitrogen, Thermo Fisher Scientific, Basel, Switzerland).

### 4.15. Quantification of KC Immunofluorescence Staining for Different Markers In Vitro

Fixed KCs were stained as described above, and the ratio of cells positive for the specific marker were quantified in a total of 100 cells using 20× magnification images. Three microscopic fields at 20× were used in each group (*n* = 6 independent donors/experiments). These images were analyzed using Photoshop 7.0 (Adobe Systems Inc., Germany).

### 4.16. Statistical Analysis

Data are shown as mean ± standard deviation (SD). Statistical comparisons between multiple groups (Control, ASC-CM, and TGF-β1) were assessed through one-way or two-way ANOVA using GraphPad Prism 9.3.1 (Graph Pad software, La Jolla, CA, USA).

* indicates a *p*-value from 0.01 to 0.05 (significant).
** indicates a *p*-value from 0.001 to 0.01 (very significant).
*** indicates that *p* < 0.001 (extremely significant).
ns = not significant (*p* > 0.05).

## 5. Conclusions

Over the last decades, many efforts have been made to develop new autologous tissue-engineered skin substitutes to replace skin defects. However, these therapies rely on the efficient numbers of patient’s cells to produce large-sized skin substitutes. In this study, we demonstrated that the ASC-CM treatment significantly enhanced the cell proliferation and migration of human keratinocytes in vitro. These two parameters, along with the enrichment of basal keratinocytes upon ASC-CM treatment, favorably impact the clinical application of those cells for cell-based skin replacement therapies. However, further in vivo animal studies are needed to confirm the positive effect of the ASC-CM on skin substitutes prior to transplantation.

## Data Availability

The data that support the findings of this study are available on request from the corresponding author.

## Supplementary Materials

The following supporting information can be downloaded at: https://www.mdpi.com/article/10.3390/ijms241914726/s1, Figure S1: Gating strategy for flow cytometry analysis.
