# Supplementary material for: Effects of an Adipose Mesenchymal Stem Cell-Derived Conditioned medium and TGF-β1 on Human Keratinocytes In Vitro
Source: Int J Mol Sci. 2023 Sep 29;24(19):14726. doi: 10.3390/ijms241914726 (PMC10572767; doi:10.3390/ijms241914726)
Supplement: Supplementary file 1 [file ijms-24-14726-s001.zip › ijms-2588213-supplementary.pdf]

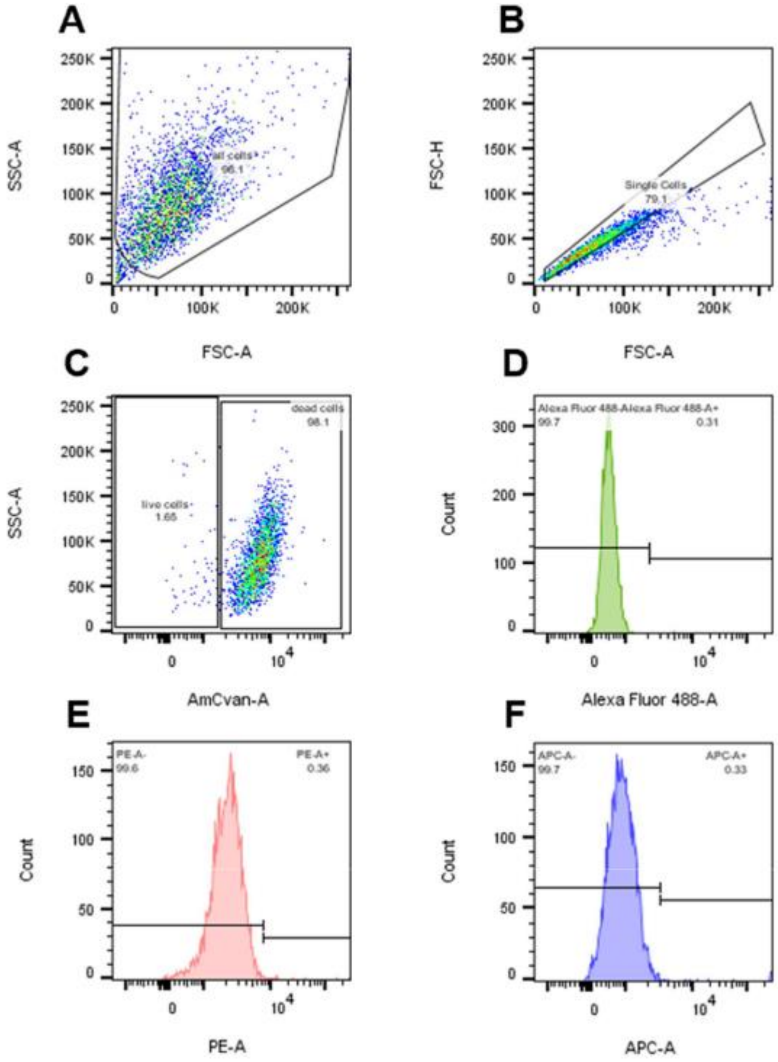

Supplementary Figure S1. Gating strategy for flow cytometry analysis. Representative dot blots from one donor. (A) The side scatter area (SSC-A) was plotted against forward scatter area (FSC-A) to select cell population of interest and to exclude cell debris. (B) Selection of single cells from the population of interest by plotting forward scatter height (FSC-H) against FSC-area (FSC-A). (C) Selection of live cells stained negative for Zombie Aqua. Here, SSC-A was plotted against the Aqua l/D channel. (D-F) Isotype controls: Alexa Fluor 488 (D), PE (E), and APC (F) were used as negative controls to differentiate non-specific background signal from specific antibody staining.
